# Predicting and Analyzing Road Traffic Injury Severity Using Boosting-Based Ensemble Learning Models with SHAPley Additive exPlanations

**DOI:** 10.3390/ijerph19052925

**Published:** 2022-03-02

**Authors:** Sheng Dong, Afaq Khattak, Irfan Ullah, Jibiao Zhou, Arshad Hussain

**Affiliations:** 1 School of Civil and Transportation Engineering, Ningbo University of Technology, Fenghua Road No. 201, Ningbo 315211, China; dongsheng@nbut.edu.cn; 2 The Key Laboratory of Road and Traffic Engineering, Ministry of Education, Tongji University, 4800 Cao’an Road, Jiading, Shanghai 201804, China; 3 Department of Civil Engineering, International Islamic University, Sector H-10, Islamabad 1243, Pakistan; irfanullah@iiu.edu.pk; 4 College of Transportation Engineering, Tongji University, 4800 Cao’an Road, Jiading, Shanghai 201804, China; zhoujibiao@tongji.edu.cn; 5 NUST Institute of Civil Engineering, National University of Sciences and Technology, Sector H-12, Islamabad 44000, Pakistan; drarshad@nit.nust.edu.pk

**Keywords:** traffic safety, road traffic injuries, boosting-based ensemble models, SHapley Additive exPlanations

## Abstract

Road traffic accidents are one of the world’s most serious problems, as they result in numerous fatalities and injuries, as well as economic losses each year. Assessing the factors that contribute to the severity of road traffic injuries has proven to be insightful. The findings may contribute to a better understanding of and potential mitigation of the risk of serious injuries associated with crashes. While ensemble learning approaches are capable of establishing complex and non-linear relationships between input risk variables and outcomes for the purpose of injury severity prediction and classification, most of them share a critical limitation: their “black-box” nature. To develop interpretable predictive models for road traffic injury severity, this paper proposes four boosting-based ensemble learning models, namely a novel Natural Gradient Boosting, Adaptive Gradient Boosting, Categorical Gradient Boosting, and Light Gradient Boosting Machine, and uses a recently developed SHapley Additive exPlanations analysis to rank the risk variables and explain the optimal model. Among four models, LightGBM achieved the highest classification accuracy (73.63%), precision (72.61%), and recall (70.09%), F1-scores (70.81%), and AUC (0.71) when tested on 2015–2019 Pakistan’s National Highway N-5 (Peshawar to Rahim Yar Khan Section) accident data. By incorporating the SHapley Additive exPlanations approach, we were able to interpret the model’s estimation results from both global and local perspectives. Following interpretation, it was determined that the Month_of_Year, Cause_of_Accident, Driver_Age and Collision_Type all played a significant role in the estimation process. According to the analysis, young drivers and pedestrians struck by a trailer have a higher risk of suffering fatal injuries. The combination of trailers and passenger vehicles, as well as driver at-fault, hitting pedestrians and rear-end collisions, significantly increases the risk of fatal injuries. This study suggests that combining LightGBM and SHAP has the potential to develop an interpretable model for predicting road traffic injury severity.

## 1. Introduction

Road traffic injuries (RTIs) are a leading cause of morbidity and death on a global scale. Understanding the sequence of events preceding a road traffic injury is critical for enhancing road safety and developing interventions that reduce the prevalence and severity of road trauma. By 2030, RTIs are expected to overtake cancer as the sixth leading cause of death worldwide [1]. Despite significant advances in traffic and transportation systems, road safety continues to be a concern, with over 1.35 million people killed and another 50 million injured in road collisions each year. Automobile accidents killed 22,441 people and injured 2.18 million people in 2015, according to the National Highway Traffic Safety Administration [2], which collects road data for all 50 states in the United States. The total number of fatal crashes in the United States increased to approximately 35,000 in 2016. Additionally, according to the Washington Annual Collision Summary (WACS), the region experienced 117,053 crashes, including 499 fatal collisions, 36,531 injury collisions, and 77,358 property-damage-only crashes, implying that a crash occurs every 4.5 min and someone dies in a crash every 16 h [3].

Traffic accidents have resulted in enormous economic, physical, and emotional distress for the families involved, as well as productivity losses for society as a whole. The World Health Organization (WHO) [4] estimates that 74% of worldwide road traffic accidents occur in low- and middle-income countries combined, making up 90% of the total global number. In addition, a large proportion of the world’s population lives in countries with a low or middle income. There are still 54% of the world’s total registered vehicles in these countries and a large proportion in relation to the number of vehicles involved in traffic accidents. The fact that death rates fell in high-income countries between 2000 and 2015, but rose in low-income countries, shows just how serious traffic issues are in developing countries.

Rapid population growth in Asia’s developing countries, particularly in South Asia, increased mobility, and increased motorized traffic on high-speed roads, all contribute to the development of RTIs. Almost no day passes without a road traffic accident on their national highways or motorways, resulting in an increasing number of injuries and fatalities and significant economic losses [5,6,7,8,9]. As is the case in other developing countries, Pakistan is increasingly confronted with serious road safety issues. Pakistan’s vehicle ownership increased by 18.3% over the last two decades, owing to a 2% annual population increase and a 3.3% increase in rapid urbanization [10]. In addition, air travel is out of reach for the majority of the population, and the potential for inland water transportation has not been fully realized. The excessive reliance on the roadway network has placed an undue strain on the National Highways. The occurrence of road traffic accidents has a substantial psychological and economic impact on both individuals and economies. It is very important to figure out and measure the factors that cause accidents in order to improve proactive highway design and accident frequency reduction.

Understanding the causes of road traffic fatalities and injuries has remained a priority, and road safety analysis has advanced significantly over the years, particularly in developing methodologies for modelling the relationship between injury severity and risk factors, gaining knowledge about the mechanism of accident occurrence, and developing safety policies and countermeasures. Over the past several decades, efforts have been made to comprehend the intrinsic relationship between collision frequency and risk variables, such as road geometry, vehicle type, collision type, seasonal effect, traffic regulation, time of day, driver characteristics, and environmental conditions. Numerous statistical models have been used to estimate the severity of traffic accident injuries. In the past, injury severity analysis and prediction have been dominated by statistical methods, such as the linear, nonlinear, generalised linear model (GLM), Poisson regression model (PRM), ordered probit model (OPM), mixed logit model (MLM), Bayesian ordered probit model (BOPM), random parameters ordered probit model (RPOPM), and cellular automata (CA) model, which were regarded as reasonable attempts at thoroughly formulating the relationship between the number of predicting variables [11,12,13,14,15,16,17,18,19,20,21,22,23,24,25]. Modelling overall accidents may not be as valuable as one might think in terms of creating safety countermeasures, as various types of accidents are frequently linked to distinct sets of primary variables. By combining all types of crashes, it may become more difficult to identify and precisely estimate the impact of a risk factor on various types of crashes, as well as to devise suitable and cost-effective safety mitigation strategies. Thus, one of the goals of this study is to determine how the varying effects of risk variables affect the severity of road traffic injuries using advanced boosting-based ensemble models.

Traditional statistical approaches, while having rigor and well-defined functional forms, frequently necessitate strict model assumptions about the relationship between independent and dependent variables [26]. The model estimation outputs may be skewed or incorrect if the assumptions are violated. Similarly, combining modern data sources frequently produces complex data sets with a large number of dimensions that are difficult to model using traditional statistical techniques. Machine learning methods, on the other hand, are highly adaptable, require few or no prior assumptions about the data, and can handle missing values, noise, and outliers [27]. In the field of traffic safety research, machine learning techniques have received a lot of attention. It has become one of the most widely used and fascinating tools for estimating the severity of injuries in road traffic accidents [28,29,30,31,32,33,34,35,36,37,38,39,40,41]. Tree-based ensemble learning models have been used in a variety of fields, including landslide susceptibility mapping [42], software bug prediction [43], and bioinformatics [44].

While high predictive performance is desirable for machine learning models, it is more critical to understand the underlying mechanism by which accidents occur, especially for the purpose of developing accident prevention countermeasures. As a result, in a number of machine learning-based traffic safety studies, sensitivity analysis was used to quantify the effects of risk factors on the severity of injuries [45,46]. To summarise, two types of sensitivity analysis exist: partial dependence plots (PDP) and local sensitivity analysis (LSA) [47]. However, both the LSA and the PDP are concerned about the assumption of independence. In practice, the assumption of independent risk variables may fail to hold true, resulting in erroneous estimates of the safety effect.

To address this issue, several researchers used the Shapley Additive exPlanations (SHAP) approach [48], which estimates the variable’ total, main, and interaction effects. Hu et al. [49], for example, utilised SHAP to assess the outcomes of a convolutional neural network (CNN) model in order to assess critical variables that affect the likelihood of crashes at road intersections. The SHAP analysis was used to identify critical factors in *E. coli* concentration data that could be used to predict beach water quality [50]. Parsa et al. [51] used SHAP to investigate the effect of various variables on the occurrence of highway accidents. Additionally, SHAP analysis is used to rank different variables in order to identify failure modes in reinforced concrete columns [52].

In terms of data, the database’s quality and breakdown dictate the statistical and machine learning approaches used and the reliability of the findings, which can assist and guide policy makers in developing strategic plans and implementing initiatives to strengthen traffic safety and thus make the roads safer. It should be noted that any approach used is constrained by the database’s limitations. Nonetheless, there is a disparity among countries, and even among local jurisdictions, in terms of data uniformity. For instance, Casado-Sanz et al. [53] conducted an international comparison of various risk variables compiled from international databases. Table 1 demonstrates an international comparison of risk variables in major databases and guidelines of various countries (USA, Australia, New Zealand, and databases, and EU directive requirements) and situates Pakistan within the context of road traffic safety.

In this study, we aim to address the underappreciated issue of model interpretability for predicting injury severity by developing predictive and transparent booting-based ensemble learning models. To accomplish this, we used and compared four boosting-based ensemble learning models: Novel Natural Gradient Boosting (NGBoost) [54], Categorical Boosting (CatBoost) [55], Light Gradient Boosting Machine (LightGBM) [56], and Adaptive Boosting (AdaBoost) [57]. Additionally, we broadened our comparison by including commonly used machine learning models in studies of traffic safety. We then used SHAP to conduct variable importance analyses on the optimal model in order to predict the most important risk variables for injury severity, assess their robustness, and dissect their interactions with other risk variables. The SHAP yields significant findings that can be used to guide the implementation of cost-effective traffic safety countermeasures.

The rest of this paper is organized as follows: The following section summarizes the study’s methodology and data, followed by descriptions of the modelling processes, namely NGBoost, CatBoost, LightGBM, and AdaBoost, as well as Shapley Additive exPlanations. Section 3 discusses the findings and the optimal model interpretation. Finally, Section 4 summarizes the findings and makes additional research recommendations.

## 2. Methodology

**Figure 1:**
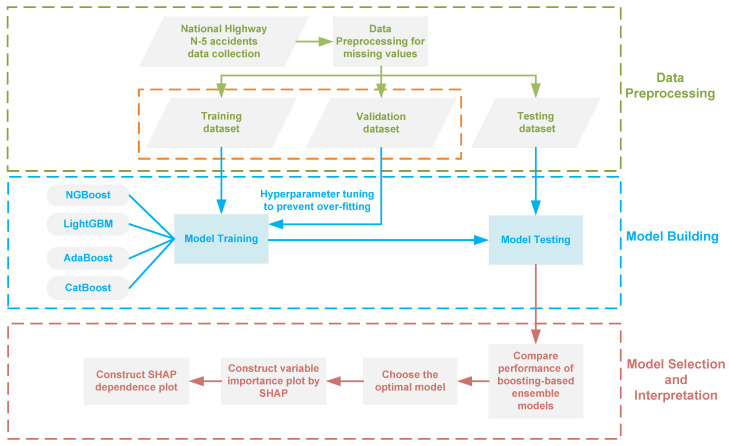
Framework of boosting-based ensemble learning models and SHAP analysis for model interpretation.

The entire operational framework proposed in this study is depicted in Figure 1. To begin with, the original accident dataset is preprocessed by eliminating redundant and erroneous information. The ensemble learning models based on boosting are initially developed on the basis of data partitioning into training, validation, and testing data sets. The training data set is used to build the classification model, the validation data set is used to fine-tune the hyperparameters of the models, and the test data set is used to evaluate the models’ performance. Once the optimal model with the best performance is identified, SHAP approach is utilized to establish additive attributes that are then employed to determine the importance of variables for injury severity and the contributions of various risk variables to each severity mode.

### 2.1. Study Route

**Figure 2:**
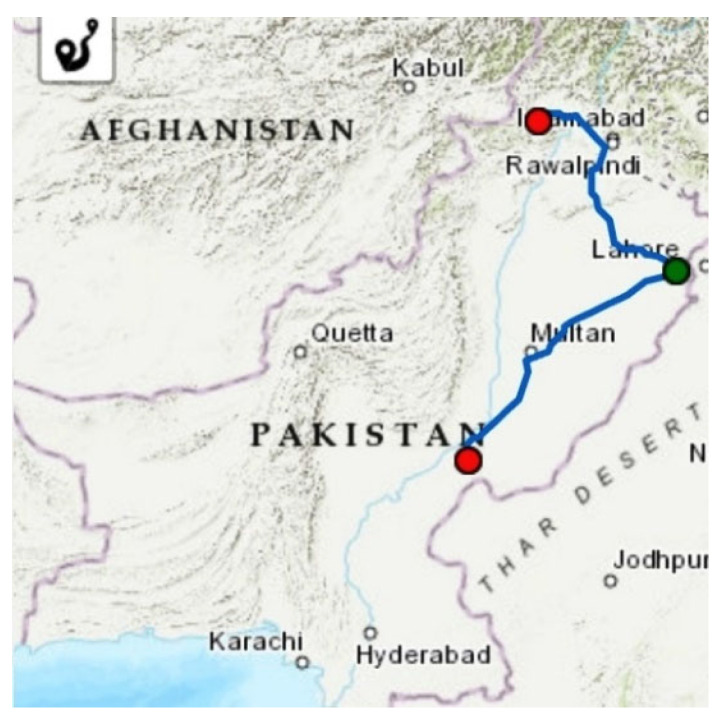
National Highway N-5 in Pakistan (Peshawar to Rahim Yar Khan Section).

The data for this study come from the records of traffic accidents that occurred on urban and rural segments of National Highway N-5 (Peshawar to Rahim Yar Khan) between 2015 and 2019. The N-5 is a two-lane divided two-way highway that connects Torkham in Pakistan’s Khyber Pakhtunkhwa province to Karachi in Sindh province, connecting major cities along its alignment as illustrated in Figure 2. It is Pakistan’s longest highway, measuring 1819 km (1310 miles) in length, and passes through three provinces: Sindh, Punjab, and Khyber Pakhtunkhwa. It is a major arterial connecting the north and south of the country and carries the most of the country’s traffic. Most heavy vehicles use this route to transport freight from Karachi’s seaport to upcountry cities. The maximum speed limit for light transport vehicles (LTV), which includes passenger cars, pickup trucks, and vans, ais 100 km per hour. The maximum speed limit for heavy transport vehicles, which includes buses, trucks, and trailers, is 90 km per hour.

### 2.2. Data Description

Pakistan’s National Highways and Motorway Police (NH & MP) are tasked with ensuring the safety and security of the country’s highways and motorways. It also keeps track of road traffic crashes by filling out a crash investigation form at the crash scene as soon as one occurs. The NH & MP utilizes a four-page crash form called “Microcomputer Accident Analysis Proforma” (MAAP) for the purpose of recording crash minutiae. The MAAP tracks each accident on National Highway N-5 using a set of twenty-two distinct variables pertaining to the environment, the crash, the roadway, the vehicle, and the drivers. Similarly, MAAP depicts injury severity levels according to four categories: fatal injury, major injury, minor injury, and no injury (property damage only (PDO)). In our study, we classified PDO, minor, and major injuries as “non-fatal injuries”, and the rest as “fatal injuries” as part of a binary classification problem. Table 2 below summarizes these variables with their description and occurrence frequencies.

### 2.3. Boosting-Based Ensemble Learning Classification Models for Injury Severity

In this research, we have employed four advanced boosting-based ensemble learning models, that is, novel Natural Gradient Boosting (NGBoost), Categorical Boosting (CatBoost), Light Gradient Boosting Machine (LightGBM) and Adaptive Boosting (AdaBoost) to accurately predict injury severity using various risk variables. Below, we explain these four boosting-based ensemble models.

#### 2.3.1. Natural Gradient Boosting (NGBoost)

**Figure 3:**
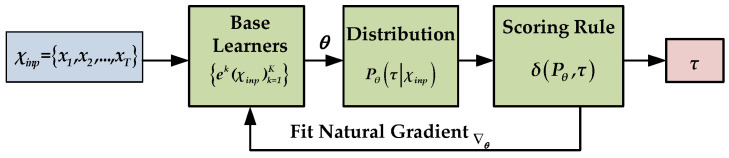
Block diagram of NGBoost.

NGBoost is a supervised natural gradient descent (NGD)-based boosting algorithm that was recently developed [54]. It can be used for classification as well as probabilistic regression [58]. It is composed of three fundamental components: a base learner, a parametric probability distribution, and a scoring rule. The conceptual framework of NGBoost is illustrated in Figure 3.

In Figure 3, $\chi_{\mathrm{inp}}$ denotes the given input risk variables, $e^{k}$ is the base learner (Decision Tree), k is the boosting iteration, τ is the predicted target, and θ denotes the target distribution parameters. The NGBoost model creates a conditional probability distribution function $\left\{P_{\theta }\left(\tau |\chi_{\mathrm{inp}}\right)\right\}$ of each predicted output in the range of 0 to 1. The higher the value of the aforementioned function, the more likely it is to correctly predict the data class, and vice versa. The framework made use of boosting to construct a series of decision trees (DTs) with minimal loss during model training. In other words, each DT learned from the preceding tree and improved the next tree in order to enhance the performance of the model. Additionally, it should be noted that this is the first paper, to our knowledge, that discusses the use of the NGBoost model for predicting the severity of road traffic injuries.

#### 2.3.2. Categorical Boosting (CatBoost)

The CatBoost is a novel version of the gradient-boosting decision tree algorithm. It has strong learning capabilities for dealing with extremely nonlinear data [55]. The Gradient Boosting Decision Tree (GBDT) algorithm combines numerous decision trees to create a high-accuracy model, and the progress can be expressed as Equation (1):

(1) $$y\left(x\right)=\sum_{t=1}^{T}f_{t}\left({x,\theta }_{t}\right)$$

where x denotes the variable vector, T denotes the number of trees, $\theta_{t}\left(t=1,2,\ldots ,T\right)$ denotes a learned parameter, and $f_{t}\left({x,\theta }_{t}\right)$ denotes the learned decision trees that are learned. Given a set of training samples $D={\left\{\left(x_{k}{,y}_{k}\right)\right\}}_{1}^{n}$, where n denotes the total number of samples in training data, $x_{k}\left(k=1,2,\ldots ,n\right)$ is the sample data points, and $y_{k}$ indicates true sample label. In order to learn the model in Equation (1), the Equation (2) objective function is required to be minimized:

(2) $$O\left(f_{t}\right)=\sum_{i=1}^{n}L\left(y_{k},{\bar{y}}_{k}\right)+\sum_{t=1}^{T}\Omega \left(f_{t}\right)$$

where ${\bar{y}}_{k}$ denotes the predicted sample label, L represent the loss function, which is actually the difference between $y_{k}$ and ${\bar{y}}_{k}$, and Ω represent the regular function, which is employed to penalize the complexity of $f_{t}$. It is defined as Equation (3):

(3) $$\Omega =\alpha q+\frac{1}{2}\beta {\left\|\omega \right\|}^{2}$$

where *α* denotes a penalty parameter, which controls the number of leaf nodes q, *β* represent the regularization parameter, and ω represents the weight coefficient. Let ζ represent the loss function negative gradient, then the objective function is minimized in the direction of ζ is given by Equation (4):

(4) $$\zeta =-\left[\frac{\partial L\left(y_{k},{\bar{y}}_{k}\right)}{\partial {\bar{y}}_{k}}\right]$$

By and large, conventional GBDT algorithms exhibit prediction offset, impairing the model’s generalization ability. CatBoost was proposed with two major enhancements to address this shortcoming [59]: (1) the ordered boosting technique was used to obtain an unbiased gradient estimation and minimise the prediction offset; (2) the oblivious tree technique has been used to improve the model’s reliability and prediction speed. Additionally, to improve the strategy’s handling of categorical variables, the greedy target-based statistics strategy was strengthened by incorporating prior terms into the CatBoost algorithm, which is composed of three major steps: (1) all sample datasets are ordered randomly; (2) similar samples are chosen and the average label for similar samples is calculated; and (3) the variables in each sample are digitised by adding the prior term and its associated weight coefficients. The strategy for optimising greedy target-based statistics is expressed in Equation (5):

(5) $$\bar{x_{k}^{i}}=\frac{\sum_{j=1}^{n}\left\{x_{j}^{i}{=x}_{k}^{i}\right\}{.y}_{i}+aP}{\sum_{j=1}^{n}\left\{x_{j}^{i}{=x}_{k}^{i}\right\}+a}$$

where $x_{k}^{i}$ denotes the kth sample’s ith category variable, ${\bar{x}}_{k}^{i}$ denotes the corresponding variable, P denotes the increased prior value, and a denotes the weight coefficient a > 0. Prior values can be used to effectively reduce noise introduced by low-frequency variables and avoid the over-fitting phenomenon.

#### 2.3.3. Light Gradient Boosting Machine (LightGBM)

**Figure 4:**
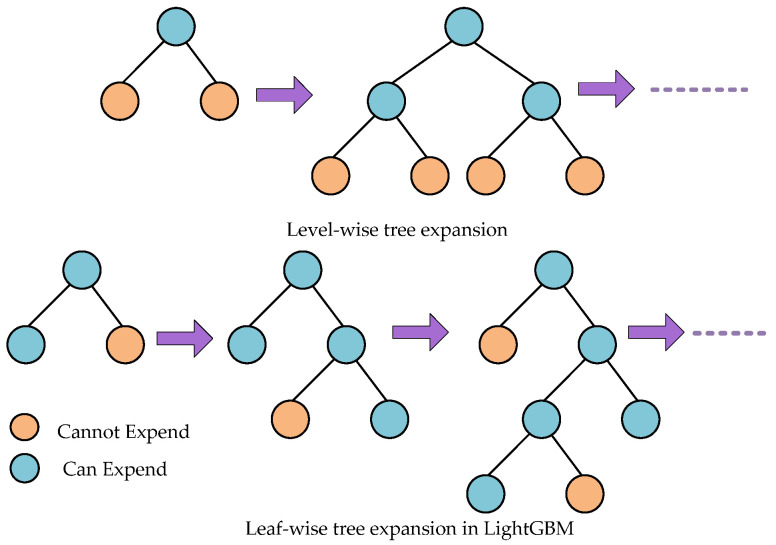
Tree expansion in LightGBM.

LightGBM is a variation of the gradient boosting decision tree (GBDT) created by Microsoft and Peking University researchers [56] to overcome the efficiency and scalability challenges associated with GBDT when used to solve problems with high-dimensional input variables and huge data sets. Because it is based on decision tree algorithms, it splits the tree leaf-wise, whereas other boosting methods divide the tree level-wise. When growing on the same leaf, the leaf-wise method reduces loss more than the level-wise strategy, resulting in much higher classification accuracy than any of the known boosting algorithms. Tree development is shown in Figure 4 for both LightGBM and severe gradient boosting. LightGBM employs two novel techniques: exclusive feature bundling (EFB) and gradient-based one-side sampling (GOSS).

Suppose a dataset with $\left\{x_{1}{,x}_{2},\ldots {,x}_{n}\right\}$ and $\left\{y_{1}{,y}_{2},\ldots {,y}_{n}\right\}$ as independent and dependent variables, respectively. The sum of the outputs of a set of decision tree models $\left(e_{t}\left(x\right)\right)$ is the predicted value of GBDT (Γ(x)), as given by Equation (6):

(6) $$\Gamma (x)=\sum_{t=1}^{T}e_{t}\left(x\right)$$

The number of decision trees is T. Finding an approximation function $\hat{\Gamma }$ that minimizes the loss function Φ(y,Γ(x)) is required to fit a GBDT model, as shown in Equation (7):

(7) $$\hat{\Gamma }=\underset{\Gamma }{\mathrm{argmin}}E_{y,S}\Phi \left(y,\Gamma (x)\right)$$

Rather than using information gain to split the internal nodes of each tree as the traditional GBDT does, LightGBM splits the internal nodes using the GOSS method. Specifically, samples with higher absolute gradient values (i.e., top α×100%) are chosen as subset A, whereas samples with lower absolute gradient values are chosen at random to form subset B (i.e., β×100%). As a result, the samples are divided based on the variance gain $V_{j}\left(d\right)$ on A∪B as Equation (8):

(8) $$V_{j}(d)=\frac{1}{n}\left[\frac{{\left(\sum_{x_{i}\in A_{1}}g_{i}+\frac{1-\alpha }{\beta }\sum_{x_{i}\in B_{1}}g_{i}\right)}^{2}}{n_{1}^{j}\left(d\right)}+\frac{{\left(\sum_{x_{i}\in A_{r}}g_{i}+\frac{1-\alpha }{\beta }\sum_{x_{i}\in B_{r}}g_{i}\right)}^{2}}{n_{r}^{j}\left(d\right)}\right]$$

where,$A_{1}=\left\{x_{i}\in {A:\;x}_{\mathrm{ij}}\leq d\right\}$, $A_{r}=\left\{x_{i}\in {A:\;x}_{\mathrm{ij}}>d\right\}$, $B_{1}=\left\{x_{i}\in {B:\;x}_{\mathrm{ij}}\leq d\right\}$, $B_{r}=\left\{x_{i}\in {B:\;x}_{\mathrm{ij}}\;>\;d\right\}$.

In each iteration, $g_{i}$ illustrates the negative gradient of the loss function for light GBM outputs. Apart from GOSS sampling, LightGBM employs the EFB technique to expedite the training process without compromising classification accuracy. Numerous applications incorporate mutually exclusive features of high-dimensional and sparse input (i.e., these features cannot be non-zero). EFB has the ability to combine these features into a single feature bundle. This algorithm can be used to make feature histograms from both these feature bundles and individual features.

In summary, LightGBM is a novel approach to ensemble learning that utilises GOSS to partition internal nodes based on variance gain and EFB to reduce the dimension of input variables. Additionally, as a decision tree-based model, LightGBM is resistant to multicollinearity. As a result, it is easy to add correlated independent variables to the LightGBM model.

#### 2.3.4. Adaptive Boosting (AdaBoost)

The AdaBoost algorithm’s basic idea is to make classifications by combining a series of weak learners using a weighted majority vote (or sum). It updates the data on a regular basis, taking into account the misclassifications of previous weak learners. This algorithm’s basic steps can be summarised as follows [57].

Given a set of training data $D={\left\{\left(x_{k}{,y}_{k}\right)\right\}}_{1}^{n}$, where n denotes the total number of data points in training data, $x_{k}\left(k=1,2,\ldots ,n\right)$ is the kth data point, and $y_{k}$ indicates true data point label. A strong classifier E(x) is generated by the following steps:

- Initially, all the data points are assigned some equal weights W i.e.,:
  $$W_{k}^{1}=\frac{1}{n}$$
- For iterations j=1,2,…,J, Train weak learner using distribution $W^{j}$, Get weak hypothesis $e^{j}$ and Select $e^{j}$ with low weighted error i.e., $\xi^{j}{=\mathrm{Pr}}_{k\sim \mathrm{Wj}}\left[e^{j}{{(x}_{k})}^{j}y_{k}\right]$. Choose $\Delta^{j}=\frac{1}{2}\ln\left(\frac{1-\xi^{j}}{\xi^{j}}\right)$.
- Update, k=1,2,…,n to obtain $W_{k}^{j+1}$ as Equation (9):
  (9) $$W_{k}^{j+1}=\frac{W_{k}^{j}\exp\left({-\Delta }^{j}y_{k}e^{j}{(x}_{k})\right)}{Z^{j}}$$
  where $Z^{j}$ is a normalization factor (chosen such that $W_{k}^{j+1}$ will be a distribution).
- After learning process and weight optimization, the final strong classifier is obtained (Equation (10)), which is based on a linear combination of all the weak classifiers:
  (10) $$E\left(x\right)=\mathrm{sign}\left(\sum_{j=1}^{J}\Delta^{j}h^{j}\left(x\right)\right)$$

### 2.4. Hyperparameter Tuning

Tuning hyperparameters is an essential step in the training of boosting-based ensemble learning models. This helps to improve the model’s generalization performance, avoid over-fitting, and reduce the complexity of the models. In this study, the classification accuracy of the model is used as a performance metric to perform tuning of hyperparameters. There are several hyperparameter tuning techniques, such as GridSearch, Random Search, and Bayesian Optimization [60]. The GridSearch and Random Search CV techniques iteratively traverse the entire space of available hyperparameter values without regard to previous results and thus become time-consuming for large parameter spaces.

In contrast, when deciding which hyperparameter set to evaluate next, Bayesian optimization considers previous evaluations. By making informed parameter combinations, it is able to focus on those areas of the parameter space that it believes will yield the most promising validation scores. This method usually requires fewer iterations to arrive at the best set of hyperparameter values [61,62]. The *HyperOpt*, which is an open-source Python library for Bayesian Optimization, was used in this research to tune the hyperparameters of four boosting-based ensemble learning models.

### 2.5. Model Evaluation

**Figure 5:**
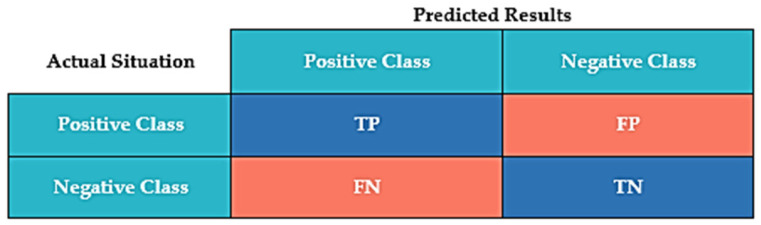
Confusion matrix plot.

The confusion matrix (contingency table) and its associated parameters (classification accuracy, precision, recall, and F1-score), as well as the area under the receiver operating characteristic (ROC) curve, are used in this study to evaluate and select the optimal ensemble model. The confusion matrix enables us to assess the performance of an ensemble model. In the confusion matrix, the predicted class instances are represented by a column, the actual class instances by a row, and the accurate prediction by the diagonal [63]. Figure 5 illustrates the confusion matrix, which can be used to calculate a variety of metrics.

The true positives (TP) and true negatives (TN) are correctly classified. A false positive (FP) occurs when an outcome is incorrectly classified as yes or positive when it is actually no or negative. When a positive result is incorrectly classified as negative, it is referred to as a false negative (FN). The TPR quantifies the proportion of positives correctly identified, whereas the FPR quantifies the proportion of negatives incorrectly classified as positives. Precision is a metric that indicates the accuracy of a classification algorithm. The precision is low, indicating a high number of FP. Recall is used to determine the completeness of a classification algorithm. A low recall indicates the presence of a large number of FN.

Equations (11)–(15) show the expression for calculating performance metrics. The expression for calculating TPR, on the other hand, is the same as for recall in Equation (13):

(11) $$\mathrm{Accuracy}=\frac{\mathrm{TP}+\mathrm{TN}}{\mathrm{TP}+\mathrm{FN}+\mathrm{TN}+\mathrm{FP}}$$

(12) $$\mathrm{Precision}=\frac{\mathrm{TP}}{\mathrm{TP}+\mathrm{FP}}$$

(13) $$\mathrm{Recall}\;\mathrm{or}\;\mathrm{TPR}=\frac{\mathrm{TP}}{\mathrm{TP}+\mathrm{FN}}$$

(14) $$F1-\mathrm{Score}=\frac{\mathrm{TP}}{\mathrm{TP}+\frac{1}{2}\left(\mathrm{FP}+\mathrm{FN}\right)}$$

(15) $$\mathrm{FPR}=\frac{\mathrm{FP}}{\mathrm{FP}+\mathrm{TN}}$$

To gain a better understanding of how well a model performed, an area under the receiver operating characteristic (AUC-ROC) curve is also used [64]. Although the confusion matrix provides a detailed analysis of the model’s performance based on predictions for each category, the AUC is sometimes preferred because it presents a comparison based on a single value. The TPR is plotted against the FPT to create the ROC. AUC values range from 0 (completely incorrect) to 1 (perfectly correct).

### 2.6. Model Interpretation

Lundberg and Lee [48] proposed SHAP (SHapley Additive exPlanations) as a new method for interpreting machine learning black-box models. This is a framework for estimating each variable’s contribution. It is based on local explanations and game theory [65].

If $x_{i,j}$ is the jth variable of the ith sample, ${\mathrm{SHAP}}_{i}$ is the model’s predicted contribution value for the ith sample, and ${\mathrm{SHAP}}_{\mathrm{base}}$ is the model’s baseline, i.e., the average value of the target variable for all samples in the model, then the SHAP value satisfies Equation (16):

(16) $${\mathrm{SHAP}}_{i}={\mathrm{SHAP}}_{\mathrm{base}}+\mathrm{shap}(x_{i,1})+\mathrm{shap}(x_{i,2}),\ldots ,\mathrm{shap}(x_{i,m})$$

where $\mathrm{shap}(x_{i,j})$ is the SHAP value of $x_{i,j}$ and m is the dimension of the variables, and ${\mathrm{shap}(x}_{i,j})$ can be thought of as the value of the jth variable in the ith sample’s contribution to the final predicted value $\bar{y}$. When ${\mathrm{shap}(x}_{i,j})$ is greater than zero, it means that this variable has a positive impact on the predicted value. If ${\mathrm{shap}(x}_{i,j})<0$ is true, the variable has a negative impact on the predicted value. The SHAP value has the advantage of reflecting not only the influence of the variable, but also their positive and negative effects. The SHAP value possesses three properties: local accuracy, absence, and consistency.

- *Local accuracy* is defined as the sum of all variable contributions equal to the model’s output. This property fulfills a fundamental requirement of the additive explanatory framework, given by Equation (17):
  (17) $${\mathrm{shap}(x)=\mathrm{SHAP}}_{\mathrm{base}}+\sum_{i=1}^{m}{\mathrm{shap}}_{j}{\times \Lambda }_{j}$$
  where ${\mathrm{shap}}_{j}$ represents the SHAP value corresponding to variable j, $\Lambda_{j}$ is an indicator function that takes the value 1 when the variable appears, and 0 otherwise.
- *Absence* i.e., the contribution of missing variable is zero. Not that a characteristic value in the structured data is empty, but that a characteristic is not observed in the sample, that is,
  $$\Lambda_{i}=0,\;\Rightarrow {\;\mathrm{SHAP}}_{i}=0$$
- *Consistency,* i.e., if the model structure alters but the degree to which a particular variable influences the output increases or remains constant, the contribution of that variable to the whole will also enhance or remain constant.

Finally, the model mapped by the sigmoid function with ${\mathrm{SHAP}}_{i}$ predicts the probability of the ith sample is given by Equation (18):

(18) $${\bar{p}}_{i}=\frac{1}{{1+e}^{{-(\mathrm{SHAP}}_{i})}}$$

where ${\bar{p}}_{i}<0.5$, then the sample classification is equal to 0, otherwise it is 1. Based on this SHAP framework, this paper explains optimal models among the four boosting-based ensemble models in terms of injury severity.

## 3. Results

In this study, four boosting-based ensemble learning models are used to predict road traffic accident injury severity. Python 3.6, a free and open-source programming language, was used for this purpose. To train the models, we used the Python packages NGBoost, CatBoost, LightGBM, and AdaBoost, as well as the Scikit-learn library. To begin with, National Highway N-5 accident data were obtained from the National Highway and Motorway Police. For model development, we partitioned the National Highway N-5 accident data into the following three subsets: 20% of the data was used for testing and withheld for model performance evaluation, while the remaining 80% was divided into training and validation subsets. The training data set was used to develop the models, and the validation data set was used to estimate the model’s performance while tuning its hyperparameters. Table 3 shows the optimal hyperparameters for four boosting-based models, obtained via Bayesian Optimization.

### 3.1. Performance Assessment

**Figure 6:**
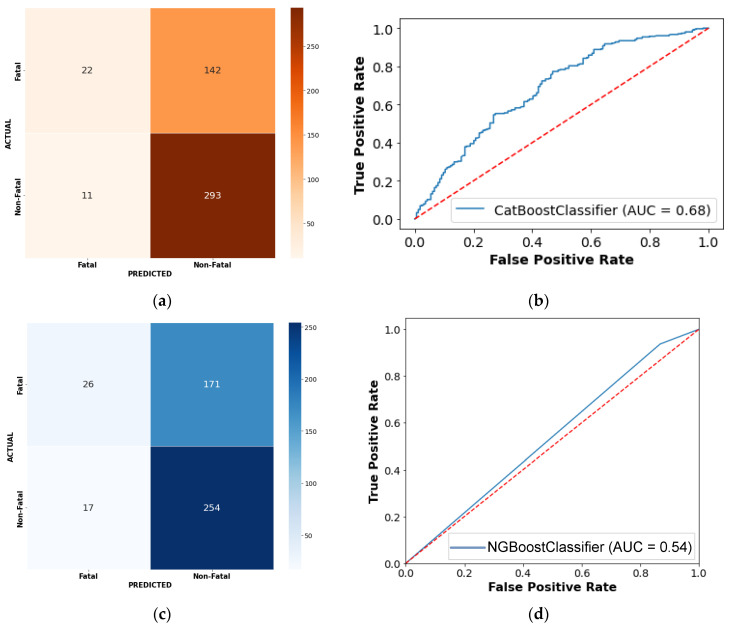

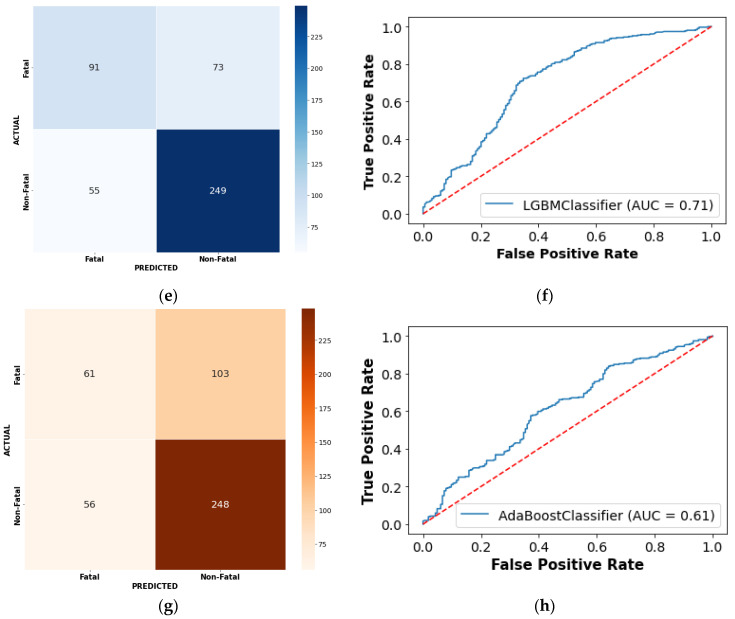
Confusion matrix of four boosting-based models using testing data: (**a**) confusion matrix by CatBoost model; (**b**) ROC curve output of CatBoost model; (**c**) confusion matrix by NGBoost model; (**d**) ROC curve output of NGBoost model; (**e**) confusion matrix by LightGBM model; (**f**) ROC curve output of LightGBM model; (**g**) confusion matrix by AdaBoost model; (**h**) ROC curve output of AdaBoost model.

Four performance indicators are used to assess the developed boosting-based ensemble models: classification accuracy, recall, precision, and F1-score. These indicators are deduced from a confusion matrix generated for binary classification problems (Figure 6). Additionally, we focused on the area under the receiver operating characteristic curve as an evaluation metric, as it is an aggregate measure. We conducted a performance comparison of the estimation results for testing dataset using the confusion matrix (Table 4) and the area under the receiver operating characteristic curve. The results of boosting-based ensemble learning models are also compared with existing models (Artificial Neural Network [66] and Logit Model [67]) that have been widely used in the analysis of road traffic injury severity.

Classification accuracy, precision, recall, F1-score, and AUC-ROC values for the CatBoost model were 67.34%, 67.32%, 55.87%, 51.28%, and 0.683, respectively, using the testing dataset. The classification accuracy, precision, recall, F1-score, and AUC-ROC values for the LightGBM model were 73.63%, 72.61%, 70.09%, 70.81%, and 0.713, respectively, using the testing dataset. The novel NGBoost model, which was used for the first time in the prediction and classification of road traffic injury severity, achieved classification accuracy (61.37%), precision (61.33%), recall (54.71%), F1-score (49.04%), and AUC-ROC (0.588), respectively. Similarly, AdaBoost model achieved classification accuracy (66.87%), precision (61.21%), recall (59.17%), F1-score (60.11%), and AUC-ROC (0.619), respectively. Comparing the performance of the models based on the confusion matrix with their corresponding matrices in Table 4 and AUC-ROC revealed that the Light GBM method provided the optimal estimation performance.

### 3.2. The Framework of Model Interpretation for Variable

#### 3.2.1. Global Variable Interpretation

Numerous methods exist for determining the relative importance of variables. Certain tree-based models, such as random forest, assign variable importance automatically, and the assignment scheme has effects on the results [68]. However, variable significance is not synonymous with variable contribution. Variable importance indicates which variable has the most significant impact on a model’s performance. Beyond identifying influential variables, the variable contributions provide an intuitive explanation for the considered output (fatal or non-fatal injuries). Two analyses are conducted in this study to determine the importance of each variable and its contribution to model estimation: the model-based variable importance approach and the SHAP summary plot approach.

**Figure 7:**
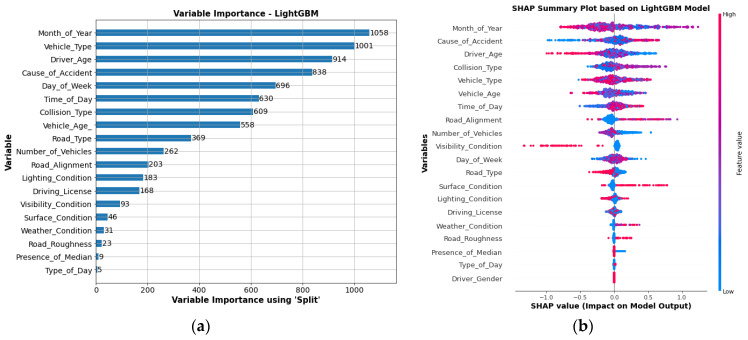
Importance plot: (**a**) variable importance based on LightGBM split criteria; (**b**) SHAP summary plot for variable importance.

The significance of each variable in the LightGBM model was first assessed. The LightGBM model’s trees are built following the steps outlined in Section 2.3.3. Let us consider the variable set $x_{1}{,x}_{2},\ldots {,x}_{m}$. The variable importance score $F_{\mathrm{imp}}{Sc}_{i}$ is then calculated as Equation (19) based on the number of times each ith variable is used to split the training data across all trees:

(19) $$F_{\mathrm{imp}}{Sc}_{i}=\left\{{s|s=W}_{i}x_{i}\right\},$$

where $W_{i}$ represents the weight of each variable, and $x_{i}$ represents the ith variable in variable set. Figure 7a shows the best variable importance score of the National Highway N-5 accidents data variable used by the LightGBM algorithm. However, the result does not indicate how much each variable contributes to the likelihood of fatal injuries.

The SHAP summary assessment was implemented to allow for a more comprehensive model analysis. From the SHAP summary plot, we estimated a quantitative value that aggregated the Shapely values and expressed the model contributions of variables (see Figure 7b). The vertical axis is used to arrange the input variables in ascending order of their influence, beginning with the most influential variable. The horizontal axis represents the SHAP value, and the color scale indicates the variable’s significance level; blue to pinkish-red indicates low to high significance. The greater the number of data points within a given range of SHAP values, the stronger the correlation between the input variable and injury severity.

As per SHAP analysis, the most important input variable in determining the injury severity is Month_of_Year, which is ranked first in the Summary Plots, followed by Cause_of_Accident, Driver_Age and Collision_Type. The significant variables are almost consistent with the variable importance obtained by the LightGBM-based model importance approach. However, the order is different due to a difference in the evaluation procedure. It is revealed that data from older drivers have negative SHAP values, which become more negative as the driver’s age increases. It confirms that drivers under the age of 30 are more likely to encounter fatal injuries. The results are consistent with past literature [69,70,71]. When comparing the months of winter to the months of spring and summer (March, June, and July), the variable Month_of_Year reveals that the majority of fatal injuries happen in the spring and summer (March, June, and July). The Cause_of_Accident, such as road surface distress, low visibility, and wrong side overtaking, are less likely to cause fatal injuries compared to bicycle rider at-fault and driver at-fault. The head-on collisions, hitting animal on road result in non-fatal injuries with SHAP values near zero or negative compared to rear-end collision, rollover and hitting obstacle on road that results in fatal injuries. Similarly, trailers and passenger cars are more prone to serious accidents.

#### 3.2.2. Local Variable Interpretation

**Figure 8:**
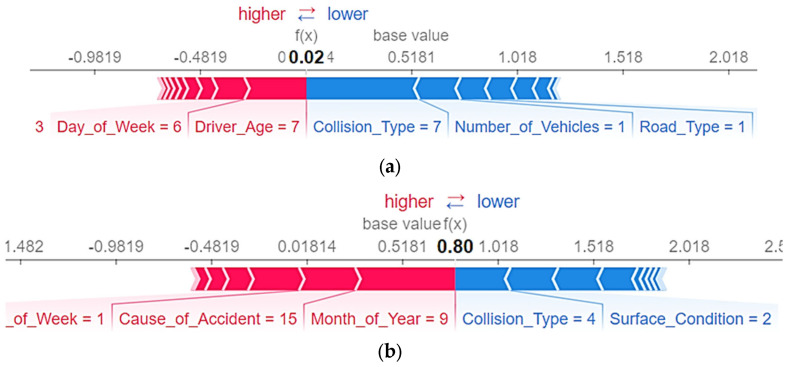
SHAP explanatory force plot: (**a**) plot for an instance value less than the base value; (**b**) plot for an instance value greater than the base value.

The SHAP explanatory force plot is shown in Figure 8 for two cases chosen at random from the actual estimate findings. On the graph, the base value (0.5181) represents the mean of the optimal LightGBM model estimations for the training data set. If the model’s output value is greater than the base value, fatal injuries occur (i.e., a lower value than the base value). When the output of the model is greater than the base value, non-fatal injuries occur (that is, a higher value than the base value). The blue arrows indicate the magnitude of the effect of input variables that increase the likelihood of fatal injuries (increased possibility of death in accidents). The effect of input variables on the occurrence of fatal injuries is indicated by red arrows (increased possibility of no death in accidents). The area occupied by variables in each arrow indicates the extent to which that variable has an effect. Consider two examples of estimated values from the training dataset that the LightGBM model correctly classified. One instance has an estimated value that is greater than the base value, while another has an estimated value that is less than the base value.

Figure 8a depicts a situation in which the estimated value (0.020) is less than the base value (0.5181). In this instance, the three variables that could potentially contribute to the fatal injuries are Driver_Age and Collision_Type. In particular, the significantly higher Driver_Age = 7 index compared to other low indexes reflects the higher expected occurrence of non-fatal injuries. Similarly, the Collision_Type = 7 index (Hit Pedestrian) reflects a higher probability of fatal injuries. Similarly, the SHAP explanatory force plot is shown in Figure 8b for another randomly selected and correctly classified instance in which the estimated value (0.800) is greater than the base value (0.5181). It demonstrates that both the month of the year and the cause of the accident played a role in predicting non-fatal injuries. To be more specific, the Month_of_Year = 9 index (September) and the Cause_of_Accident = 3 index (Tire burst) reflect a lower number of fatal injuries. However, Collision_Type = 4 index (rollover) are more prone to the occurrence of fatal injuries.

#### 3.2.3. Variable Interaction Analysis

**Figure 9:**
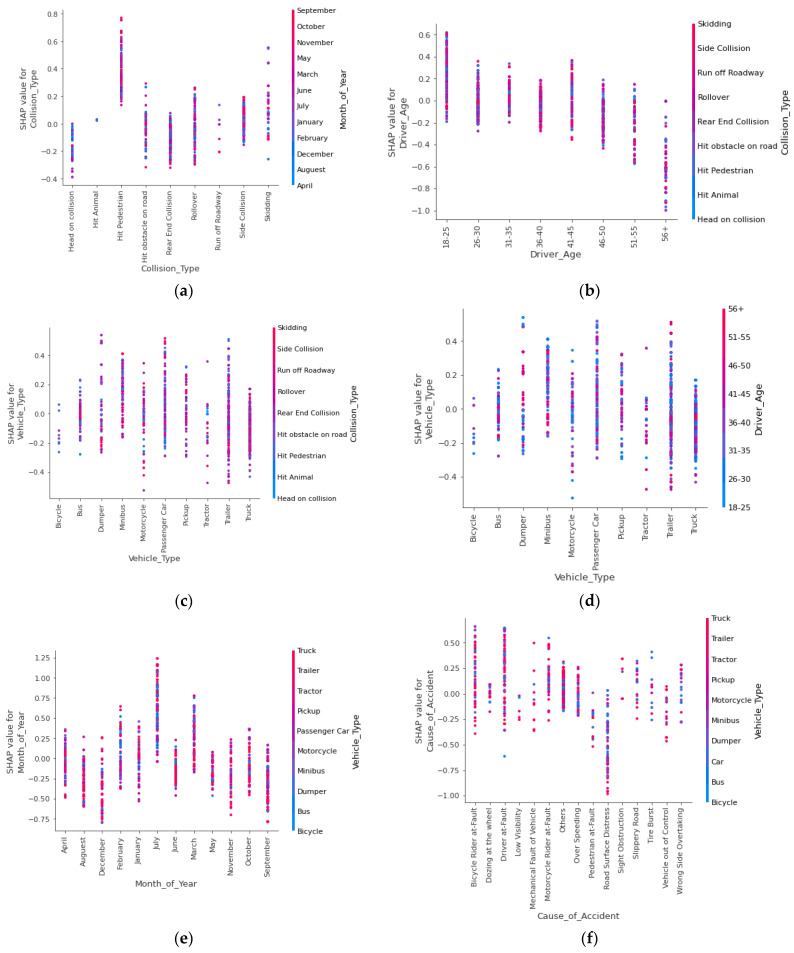
SHAP interaction plots: (**a**) impact of Collision_Type and Month_of_Year on model output; (**b**) Driver_Age and Collision_Type on model output; (**c**) impact of Vehicle_Type and Collision_Type on model output; (**d**) impact of Driver_Age and Vehicle_Type on model output; (**e**) impact of Month_of_Year and Vehicle_Type on model output; (**f**) impact of Vehicle_Type and Cause_of_Accident on model output.

The SHAP interaction plots were analysed to determine how the input variables used to estimate the optimal LightGBM model interacted in terms of their contributions (see Figure 9). The interaction analysis examined trends in Month_of_Year, Cause_of_Accident, Driver_Age, Collision_Type and Vehicle_Type. Nonetheless, other variable interactions could be evaluated as well. In Figure 9a, the scatter plots of the red and blue points illustrate the fluctuation in the Collision_Type and Collision_Type SHAP values. The SHAP value for Collision_Type is greater when Collision_Type = Hit Pedestrian, and the pattern is consistent throughout the year. This is self-evident, as the majority of locations along National Highway N-5 lack designated pedestrian crossings, increasing the likelihood that pedestrians will be struck by vehicles when they recklessly cross the road. In the majority of cases, jaywalking results in deadly injuries.

Young drivers are more likely to sustain fatal injuries than older drivers. However, drivers over the age of 50 are more likely to be involved in non-fatal crashes (Figure 9b). Young drivers are more prone to be distracted by various activities, such as ringing cell phones, texting, using a GPS device and listening to music, and these activities are connected with decreased driving performance, increased driver reaction times, decreased vehicle control, and an increased chance of a crash. Additionally, older drivers have more experience and are more likely to have non-fatal injuries. There is a possibility that young drivers are more prone to fatal injuries because they engage in risky behaviour, such as over speeding and not obeying traffic signals and signs. Sometimes, they cannot perceive the potential hazards in the surroundings and choose incorrect behaviour.

The scatter plot of Vehicle_Type and the SHAP value for Vehicle_Type are shown in Figure 9c. It demonstrates that the majority of Vehicle_Type = Trailers and Passenger Cars are more likely to result in fatal injuries as a result of rear-end crashes, rollovers, or hitting pedestrians. The trailer’s involvement in the majority of fatal injuries could be explained by the fact that National Highway N-5 is used for long-haul journeys by large trailers transporting goods from Karachi’s seaport to the countryside. Speed limit violations, braking failure, overloading, and driver distraction due to inattention or dozing can all contribute to trailer rollover accidents. Surprisingly, as indicated by the lower SHAP value, motorcycle accidents due to skidding result in non-fatal injuries. Surprisingly, the age group 26–30 is more likely to suffer fatal injuries while driving heavy vehicles compared to driving light vehicles (Figure 9d), consistent with a previous study [72].

The SHAP value for Month_of_Year = July is relatively high in the graph, showing that the majority of serious accidents with fatal injuries for heavier vehicles, such as trucks, trailers, and tractors, occur during July (Figure 9e). This result is also in line with past literature [73,74], which also highlighted that summer is more prone to crashes due to more vehicles traveling. However, in Pakistan this could be because National Highway N-5 is a major arterial that traverses through the Punjab region of Pakistan, which is known for its foggy winters, heavy monsoon precipitation, and sweltering summers. July has a maximum temperature of about 45 °C and sometimes reaches 50 °C. Increased tire pressures may result in a tire burst during July’s severe heat. The monsoon season’s torrential downpours in July may contribute to severe injuries due to wet pavements that reduce a heavy vehicle’s traction and manoeuvrability. Similarly, with Month_of_Year = September, the SHAP value is relatively low. Additionally, one can also note that truck, trailer, and tractor accidents on National Highway N-5 are nearly evenly distributed throughout the year in compared other Vehicle_Type.

Additionally, Figure 9f provides valuable insight into the involvement of Vehicle_Type in accidents and the causes of those accidents. When Bicycle Rider at-Fault and Driver at-Fault (such as wrong side overtake, traffic signal violation etc.) of trailers and passenger cars are more likely to be involved in fatal collisions The reason for this might be that bicycle riders in Pakistan do not wear helmets and often ride in the wrong direction due to the absence of bicycle-specific traffic control regulations.

## 4. Conclusions

In this research, boosting-based ensemble learning models were used in conjunction with SHAP analysis to identify critical risk variables and quantify their effects on the severity of road traffic accident injuries using the National Highway N-5 accident dataset (2015–2019). Accurate models typically capture a complete picture of the underlying relationship between injury severity and risk factors. In this research, LightGBM outperforms CatBoost, novel NGBoost, AdaBoost as well as widely used ANN and logit model in terms of predictive classification accuracy, precision, recall, F1-Score, and AUC. The newly introduced LightGBM model provides another viable option for modelling injury severity.

The lack of transparency and interpretability of machine learning models is frequently chastised. This has an effect on the widespread acceptance of models for modelling in traffic and transportation safety, although these models are more flexible and frequently more accurate than traditional predictive methods. To address the interpretability issue associated with LightGBM, the SHAP analysis was used to estimate its output in order to identify significant risk variables and quantify their impact on injury severity. The SHAP analysis’s results can be used to rank a risk variable’s overall significance. More importantly, they can be used to investigate both the individual effects of risk variables (e.g., how specific impacts may fluctuate in response to changes in the risk variable’s value) and their interaction effects.

The analysis revealed that the top four important variables that are more likely to affect injury severity are Month_of_Year, Cause_of_Accident, Driver_Age and Collision_Type. However, Type_of_Day and Work_Zone variables have the most negligible impact on the injury severity. Young drivers are more likely than older drivers to sustain fatal injuries. Improved young driver education programs, stricter driving requirements, stricter driving tests, and equipping parents with sufficient knowledge to train and educate drivers could all contribute to a reduction in the overall fatal crash rate. The month of July accounts for a large number of fatal injuries, while September accounts for a greater number of non-fatal injuries. The rainy season in the Punjab region, in addition to the high temperatures of July, may account for tire bursts and skidding, probably due to a reduction in tire-to-wet pavement friction. Extra care in driving is required during the rainy days of the monsoon (July to September) with low vehicle speed and care for runoff. It has also been observed that weekend crashes are more likely to cause fatal injuries. This finding may be due to the high traffic volume on National Highway N-5. People mostly travel to their nearby hometowns or villages on weekends, causing heavy congestion. In addition, people also travel to Sunday markets on weekends. In some cases, heavy vehicles, such as trailers, are involved in fatal crashes when drivers are drowsy at the wheel or, in some cases, crashes with bicycles. There is a need to provide bicycle-specific traffic regulations on National Highway N-5. Moreover, helmet usage should be made compulsory.

The strategy outlined in this paper can be used to conduct a large-scale analysis of traffic accidents and serves as a useful tool for policymakers and researchers engaged in traffic safety. This paper discussed only injury severity as determined by boosting ensemble in conjunction with SHAP analysis. Additional research could be conducted by using various other machine learning techniques with various additional risk factors. Additionally, future research could expand the dataset to increase its accuracy, and data-balancing techniques, such as SMOTE and ADAYSN, could be introduced to treat imbalanced data.

## Figures and Tables

**Table 1:** International comparison of risk variables from databases and guidelines of various countries with Pakistan.

Variables	EU Directive	USA	Australia	New Zealand	Pakistan
**Crash Location**	Precise as possible location	Road name, GPS coordinates	Road name, reference point, distance, direction	Road name, GPS coordinates	District and kilometer marker no. starting from Karachi city (00)
**Crash Narrative**	No	No	Yes	No	No
**Crash Sketch**	No	No	Yes, restricted access	Yes	No
**Crash Type**	Yes	Recorded in the traffic units Section	Yes	Yes	Yes
**Collision Type**	Yes	8 descriptors	Yes	Yes	Yes
**Contributing Circumstances**	No	Environmental circumstances	Yes	Yes	Yes
**Weather Conditions**	Yes	10 descriptors	Yes	5 descriptors	3 descriptors
**Lighting Condition**	Yes	7 descriptors	Yes	7 descriptors	3 descriptors
**Reported Crashes**	Not specified	All severities	All injury severities	All severities	All severities
**Definition Non-fatal Injury Levels**	Severe and non-severe injuries	Suspected serious injury, suspected minor injury, possible injury	Injured, admitted to hospital injured, required medical treatment	Major and minor injuries	Fatal injury, major injury, minor injury, and no injury
**Contributing Circumstances**	No	11 descriptors	No	Numerous causes	Yes
**Speed Limit**	Yes	Yes	Yes	Yes	No
**Surface Conditions**	Yes	10 descriptors	Yes	3 descriptors	Yes
**Road Curves**	No	Yes	Yes	4 descriptors	Yes
**Gradient**	No	Yes	No	No	No
**Age**	Yes	Date of birth	Yes	Yes	Yes
**Gender**	Yes	Yes	Yes	Yes	Yes
**Nationality**	Yes	No	Foreign drivers’ identification	Foreign drivers’ identification	No
**Alcohol Level**	Yes	Yes	Yes	Yes	No
**Drug Test Results**	No	Yes	Yes	Yes	No
**Safety Equipment**	Yes	Yes	Yes	Yes	No
**Curve Radius**	No	Yes	Yes	Yes	No
**Curve Length**	No	Yes	Yes	Yes	No

**Table 2:** Description of variables in the National Highway N-5 accidents dataset.

Type of Variable	Variable	Description	Marginal Frequency (%)
**Injury** **Severity**	Injury Severity Category	Fatal/Non-Fatal	38.09/61.91
**Vehicle** **Specific**	Type_of_Vehicle	Rickshaw/Motorcycle/Bicycle/ Car/Pickup/Minibus/Bus/Truck/ Dumper/Trailer/Tractor	5.34/6.78/10.77/ 12.69/3.50/9.19/8.48/ 20.08/4.43/16.44/2.30
	Vehicle_Age (years)	0–10/11–20/21–30/31–40/41+	32.01/36.49/15.30/9.30/6.90
	Number_of_Vehicles (Number of vehicles in crash)	Single/Multiple	33.46/66.54
**Driver** **Specific**	Driver Gender	Female/Male	0.001/99.99
	Driver_Age (Years)	18–25/26–30/31–35/36–40/41–45/46–50/51–55/55+	18.18/16.83/14.90/14.58/ 13.62/10.92/5.84/5.14
	Driving_License	No/Yes	46.52/53.48
**Environment** **Specific**	Lighting_Condition	Daylight/Night with Road Lights/Night without Road Lights	69.11/5.33/25.56
	Weather_Condition	Sunny/Cloudy/Rainy	89.85/3.59/6.56
	Visibility_Condition	Clear/Fog/Smog	96.41/3.08/0.50
**Temporal** **Specific**	Month_of_Year	January/February/March/ April/May/June/July/August/ September/October/November/ December	5.65/6.29/10.08/8.73/5.27/ 6.87/14.96/10.08/13.17/ 6.10/7.32/5.46
	Day_of_Week	Monday/Tuesday/Wednesday/ Thursday/Friday/Saturday/Sunday	10.76/12.67/14.52/13.51/16.87/ 16.82/14.85
	Time_of_Day	12:00:00 a.m.–3:59:59 a.m./4:00:00 a.m.–7:59:59 a.m./8:00:00 a.m.–11:59:59 p.m./12:00:00 p.m.–3:59:59 p.m./4:00:00 p.m.–7:59:59 p.m./8:00:00 p.m.–11:59:59 p.m.	8.97/14.41/23.09/21.13/ 21.02/11.38
	Type_of_Day	Weekday/Weekend	68.22/31.78
**Roadway** **Specific**	Alignment	Straight/Horizontal Curve/Vertical Curve/Both Horizontal and Vertical Curves	84.36/5.66/4.43/5.55
	Presence_of_Shoulder	No/Yes	2.63/97.37
	Surface_Condition	Dry/Wet	92.49/7.51
	Pavement_Roughness	Smooth/Rough/Potholes	94.23/2.52/3.25
	Road_Type	Urban/Rural	52.86/47.14
	Presence_of_Median	No/Yes	3.64/96.36
	Work_Zone	No/Yes	98.64/1.35
**Crash** **Specific**	Collision_Type	Head on Collision/Rear End Collision/Side Collision/Rollover/ Skidding/Hit Obstacle on Road/Hit Pedestrian/Hit Animal/Run off Roadway/Hitting Nearby Trees/Fell off Bridge	5.21/43.55/19.17/12.44/ 3.08/4.88/10.31/0.28/ 0.78/0.11/0.17
	Cause_of_Accident	Bicycle Rider at-Fault/Wrong Side Overtaking/Pedestrian at-Fault/Pavement Distress/Driver at-Fault/Dozing at the Wheel/Over Speeding/Motorcycle Rider at-Fault/Low Visibility/Mechanical Fault of Vehicle/Sight Obstruction/Slippery Road/Vehicle out of Control/Other	0.56/1.46/7.29/1.51/56.331.40/3.87/3.14/0.39/7.74/ 1.79/2.35/0.90/2.35/8.91

**Table 3:** Hyperparameters tuning of boosting-based ensemble models.

Algorithm	Evaluation Metric	Hyperparameters	Range	Optimal Values
CatBoost	Classification accuracy	*n*_estimators	(100, 5000)	11,600
		max_depth	(0, 10)	5
		learning_rate	(0.001, 0.5)	0.002
LightGBM	Classification accuracy	*n*_estimators	(100, 5000)	3300
		learning_rate	(0.001, 0.5)	0.042
		max_depth	(0, 10)	6
		lambda_l1	(1 × 10^−8^, 10)	0.52
		lambda_l2	(1 × 10^−8^, 10)	0.2
NGBoost	Classification accuracy	learning_rate	(0.001, 0.50)	0.01
		*n*_estimators	(100, 5000)	600
		Max_depth	(0, 10)	4
AdaBoost	Classification accuracy	*n*_estimators	(100–5000)	800
		Learning_rate	(0.01, 1)	0.5

**Table 4:** Comparison of prediction performance of various models.

Performance Metrics	Proposed Boosting-Based Ensemble Models				Existing Models for Road Traffic Injury Severity	
	CatBoost	LightGBM	NGBoost	AdaBoost	ANN [66]	Logit Model [67]
**Accuracy (%)**	67.34	73.63	61.13	66.87	62.17	60.47
**Precision (%)**	67.32	72.61	61.33	61.21	62.37	88.71
**Recall (%)**	55.87	70.09	54.71	59.17	60.60	63.10
**F1—Score (%)**	51.28	70.81	49.04	60.11	50.81	50.81
**AUC**	0.684	0.713	0.588	0.619	0.601	0.533

## Data Availability

The corresponding author will make available, upon reasonable request, the data that support the findings of this study.

## References

[1] Chekijian S., Paul M., Kohl V.P., Walker D.M., Tomassoni A.J., Cone D.C., Vaca F.E. (2014). The global burden of road injury: Its relevance to the emergency physician. Emerg. Med. Int..

[2] NHTSA (2016). 2015 motor vehicle crashes: Overview. Traffic Saf. Facts: Res. Note.

[3] Washington Annual Collision Summary 2015. https://www.wsdot.wa.gov/mapsdata/crash/pdf/2015_Annual_Collision_Summary.pdf.

[4] WHO Global Status Report on Road Safety 2015 (Report No. 9789241565066). https://apps.who.int/iris/handle/10665/189242.

[5] Hamim O.F., Hoque M.S., McIlroy R.C., Plant K.L., Stanton N.A. (2020). A sociotechnical approach to accident analysis in a low-income setting: Using Accimaps to guide road safety recommendations in Bangladesh. Saf. Sci..

[6] Hussain M., Shi J., Batool Z. (2022). An investigation of the effects of motorcycle-riding experience on aberrant driving behaviors and road traffic accidents-A case study of Pakistan. Int. J. Crashworthiness.

[7] Islam M.A., Dinar Y. (2021). Evaluation and spatial analysis of road accidents in Bangladesh: An emerging and alarming issue. Transp. Dev. Econ..

[8] Vipin N., Rahul T. (2021). Road traffic accident mortality analysis based on time of occurrence: Evidence from Kerala, India. Clin. Epidemiol. Glob. Health.

[9] Zeng Q., Hao W., Lee J., Chen F. (2020). Investigating the Impacts of Real-Time Weather Conditions on Freeway Crash Severity: A Bayesian Spatial Analysis. Int. J. Environ. Res. Public Health.

[10] Ministry of Finance (G.o.P.) Pakistan Economic Survey 2015–16. https://www.finance.gov.pk/survey_1516.html.

[11] Ma J., Kockelman K.M., Damien P. (2008). A multivariate Poisson-lognormal regression model for prediction of crash counts by severity, using Bayesian methods. Accid. Anal. Prev..

[12] Aguero-Valverde J., Jovanis P.P. (2009). Bayesian multivariate Poisson lognormal models for crash severity modeling and site ranking. Transp. Res. Rec..

[13] Nowakowska M. (2010). Logistic models in crash severity classification based on road characteristics. Transp. Res. Rec..

[14] Pei X., Wong S., Sze N.-N. (2011). A joint-probability approach to crash prediction models. Accid. Anal. Prev..

[15] Haleem K., Abdel-Aty M. (2010). Examining traffic crash injury severity at unsignalized intersections. J. Saf. Res..

[16] Chen F., Chen S. (2011). Injury severities of truck drivers in single-and multi-vehicle accidents on rural highways. Accid. Anal. Prev..

[17] Chen S., Chen F., Wu J. (2011). Multi-scale traffic safety and operational performance study of large trucks on mountainous interstate highway. Accid. Anal. Prev..

[18] Ye F., Lord D. (2011). Investigation of effects of underreporting crash data on three commonly used traffic crash severity models: Multinomial logit, ordered probit, and mixed logit. Transp. Res. Rec..

[19] Chen F., Ma X., Chen S. (2014). Refined-scale panel data crash rate analysis using random-effects tobit model. Accid. Anal. Prev..

[20] Xi J.-F., Liu H.-Z., Cheng W., Zhao Z.-H., Ding T.-Q. (2014). The model of severity prediction of traffic crash on the curve. Math. Probl. Eng..

[21] Ahmadi A., Jahangiri A., Berardi V., Machiani S.G. (2020). Crash severity analysis of rear-end crashes in California using statistical and machine learning classification methods. J. Transp. Saf. Secur..

[22] Chen F., Song M., Ma X. (2019). Investigation on the injury severity of drivers in rear-end collisions between cars using a random parameters bivariate ordered probit model. Int. J. Environ. Res. Public Health.

[23] Chen F., Ma X., Chen S., Yang L. (2016). Crash frequency analysis using hurdle models with random effects considering short-term panel data. Int. J. Environ. Res. Public Health.

[24] Chen F., Chen S., Ma X. (2016). Crash frequency modeling using real-time environmental and traffic data and unbalanced panel data models. Int. J. Environ. Res. Public Health.

[25] Marzoug R., Lakouari N., Ez-Zahraouy H., Téllez B.C., Téllez M.C., Villalobos L.C. (2022). Modeling and simulation of car accidents at a signalized intersection using cellular automata. Phys. A Stat. Mech. Appl..

[26] Alarifi S.A., Abdel-Aty M., Lee J. (2018). A Bayesian multivariate hierarchical spatial joint model for predicting crash counts by crash type at intersections and segments along corridors. Accid. Anal. Prev..

[27] Al-Moqri T., Haijun X., Namahoro J.P., Alfalahi E.N., Alwesabi I. (2020). Exploiting Machine Learning Algorithms for Predicting Crash Injury Severity in Yemen: Hospital Case Study. Appl. Comput. Math..

[28] Wen X., Xie Y., Wu L., Jiang L. (2021). Quantifying and comparing the effects of key risk factors on various types of roadway segment crashes with LightGBM and SHAP. Accid. Anal. Prev..

[29] Tang J., Zheng L., Han C., Yin W., Zhang Y., Zou Y., Huang H. (2020). Statistical and machine-learning methods for clearance time prediction of road incidents: A methodology review. Anal. Methods Accid. Res..

[30] Arteaga C., Paz A., Park J. (2020). Injury severity on traffic crashes: A text mining with an interpretable machine-learning approach. Saf. Sci..

[31] Assi K., Rahman S.M., Mansoor U., Ratrout N. (2020). Predicting crash injury severity with machine learning algorithm synergized with clustering technique: A promising protocol. Int. J. Environ. Res. Public Health.

[32] Taamneh S., Taamneh M.M. (2021). A machine learning approach for building an adaptive, real-time decision support system for emergency response to road traffic injuries. Int. J. Inj. Control. Saf. Promot..

[33] Wahab L., Jiang H. (2020). Severity prediction of motorcycle crashes with machine learning methods. Int. J. Crashworthiness.

[34] Yahaya M., Fan W., Fu C., Li X., Su Y., Jiang X. (2020). A machine-learning method for improving crash injury severity analysis: A case study of work zone crashes in Cairo, Egypt. Int. J. Inj. Control. Saf. Promot..

[35] Mohanta B.K., Jena D., Mohapatra N., Ramasubbareddy S., Rawal B.S. (2022). Machine learning based accident prediction in secure iot enable transportation system. J. Intell. Fuzzy Syst..

[36] Sangare M., Gupta S., Bouzefrane S., Banerjee S., Muhlethaler P. (2021). Exploring the forecasting approach for road accidents: Analytical measures with hybrid machine learning. Expert Syst. Appl..

[37] Topuz K., Delen D. (2021). A probabilistic Bayesian inference model to investigate injury severity in automobile crashes. Decis. Support. Syst..

[38] Worachairungreung M., Ninsawat S., Witayangkurn A., Dailey M.N. (2021). Identification of Road Traffic Injury Risk Prone Area Using Environmental Factors by Machine Learning Classification in Nonthaburi, Thailand. Sustainability.

[39] Wu P., Meng X., Song L. (2020). A novel ensemble learning method for crash prediction using road geometric alignments and traffic data. J. Transp. Saf. Secur..

[40] Jiang L., Xie Y., Wen X., Ren T. (2020). Modeling highly imbalanced crash severity data by ensemble methods and global sensitivity analysis. J. Transp. Saf. Secur..

[41] Peng H., Ma X., Chen F. (2020). Examining Injury Severity of Pedestrians in Vehicle–Pedestrian Crashes at Mid-Blocks Using Path Analysis. Int. J. Environ. Res. Public Health.

[42] Pham B.T., Nguyen-Thoi T., Qi C., Van Phong T., Dou J., Ho L.S., Van Le H., Prakash I. (2020). Coupling RBF neural network with ensemble learning techniques for landslide susceptibility mapping. Catena.

[43] Pandey S.K., Mishra R.B., Tripathi A.K. (2020). BPDET: An effective software bug prediction model using deep representation and ensemble learning techniques. Expert Syst. Appl..

[44] Che D., Liu Q., Rasheed K., Tao X. (2011). Decision tree and ensemble learning algorithms with their applications in bioinformatics. Softw. Tools Algorithms Biol. Syst..

[45] García-Herrero S., Gutiérrez J.M., Herrera S., Azimian A., Mariscal M. (2020). Sensitivity analysis of driver’s behavior and psychophysical conditions. Saf. Sci..

[46] Jiang L., Xie Y., Ren T. Modelling highly unbalanced crash injury severity data by ensemble methods and global sensitivity analysis. Proceedings of the Transportation Research Board 98th Annual Meeting.

[47] Cattarin G., Pagliano L., Causone F., Kindinis A., Goia F., Carlucci S., Schlemminger C. (2018). Empirical validation and local sensitivity analysis of a lumped-parameter thermal model of an outdoor test cell. Build. Environ..

[48] Lundberg S.M., Lee S.-I. A unified approach to interpreting model predictions. Proceedings of the 31st international conference on neural information processing systems.

[49] Hu J., Huang M.-C., Yu X. (2020). Efficient mapping of crash risk at intersections with connected vehicle data and deep learning models. Accid. Anal. Prev..

[50] Li L., Qiao J., Yu G., Wang L., Li H.-Y., Liao C., Zhu Z. (2022). Interpretable tree-based ensemble model for predicting beach water quality. Water Res..

[51] Parsa A.B., Movahedi A., Taghipour H., Derrible S., Mohammadian A.K. (2020). Toward safer highways, application of XGBoost and SHAP for real-time accident detection and feature analysis. Accid. Anal. Prev..

[52] Mangalathu S., Hwang S.-H., Jeon J.-S. (2020). Failure mode and effects analysis of RC members based on machine-learning-based SHapley Additive exPlanations (SHAP) approach. Eng. Struct..

[53] Casado-Sanz N., Guirao B., Attard M. (2020). Analysis of the risk factors affecting the severity of traffic accidents on Spanish crosstown roads: The driver’s perspective. Sustainability.

[54] Duan T., Anand A., Ding D.Y., Thai K.K., Basu S., Ng A., Schuler A. Ngboost: Natural gradient boosting for probabilistic prediction. Proceedings of the International Conference on Machine Learning.

[55] Hancock J.T., Khoshgoftaar T.M. (2020). CatBoost for big data: An interdisciplinary review. J. Big Data.

[56] Chen T., Xu J., Ying H., Chen X., Feng R., Fang X., Gao H., Wu J. (2019). Prediction of extubation failure for intensive care unit patients using light gradient boosting machine. IEEE Access.

[57] Wang F., Jiang D., Wen H., Song H. (2019). Adaboost-based security level classification of mobile intelligent terminals. J. Supercomput..

[58] Kavzoglu T., Teke A. (2022). Predictive Performances of Ensemble Machine Learning Algorithms in Landslide Susceptibility Mapping Using Random Forest, Extreme Gradient Boosting (XGBoost) and Natural Gradient Boosting (NGBoost). Arab. J. Sci. Eng..

[59] Yuan Z., Zhou T., Liu J., Zhang C., Liu Y. (2021). Fault Diagnosis Approach for Rotating Machinery Based on Feature Importance Ranking and Selection. Shock Vib..

[60] Liang S., Peng J., Xu Y., Ye H. (2021). Passive Fetal Movement Recognition Approaches Using Hyperparameter Tuned LightGBM Model and Bayesian Optimization. Comput. Intell. Neurosci..

[61] Xia Y., Liu C., Li Y., Liu N. (2017). A boosted decision tree approach using Bayesian hyper-parameter optimization for credit scoring. Expert Syst. Appl..

[62] Turner R., Eriksson D., McCourt M., Kiili J., Laaksonen E., Xu Z., Guyon I. (2021). Bayesian optimization is superior to random search for machine learning hyperparameter tuning: Analysis of the black-box optimization challenge 2020. arXiv.

[63] Brownlee J. (2016). Machine Learning Algorithms from Scratch with Python.

[64] Sun F., Dubey A., White J. DxNAT—Deep neural networks for explaining non-recurring traffic congestion. Proceedings of the 2017 IEEE International Conference on Big Data (Big Data).

[65] Merrick L., Taly A. (2019). The explanation game: Explaining machine learning models with cooperative game theory. arXiv.

[66] Shaik M.E., Islam M.M., Hossain Q.S. (2021). A review on neural network techniques for the prediction of road traffic accident severity. Asian Transp. Stud..

[67] Mujalli R.O., Oña J.d. (2013). Injury severity models for motor vehicle accidents: A review. Proc. Inst. Civ. Eng. Transp..

[68] Kang K., Ryu H. (2019). Predicting types of occupational accidents at construction sites in Korea using random forest model. Saf. Sci..

[69] Zhang C., He J., Wang Y., Yan X., Zhang C., Chen Y., Liu Z., Zhou B. (2020). A crash severity prediction method based on improved neural network and factor Analysis. Discret. Dyn. Nat. Soc..

[70] Al Reesi H., Al Maniri A., Adawi S.A., Davey J., Armstrong K., Edwards J. (2016). Prevalence and characteristics of road traffic injuries among young drivers in Oman, 2009–2011. Traffic Inj. Prev..

[71] Donmez B., Liu Z. (2015). Associations of distraction involvement and age with driver injury severities. J. Saf. Res..

[72] Behnood A., Al-Bdairi N.S.S. (2020). Determinant of injury severities in large truck crashes: A weekly instability analysis. Saf. Sci..

[73] Ullah H., Farooq A., Shah A.A. (2021). An Empirical Assessment of Factors Influencing Injury Severities of Motor Vehicle Crashes on National Highways of Pakistan. J. Adv. Transp..

[74] Hao W., Kamga C., Wan D. (2016). The effect of time of day on driver’s injury severity at highway-rail grade crossings in the United States. J. Traffic Transp. Eng..

